# Supplementary figures and images for: The biomechanics of chewing and suckling in the infant: A potential mechanism for physiologic metopic suture closure
Source: PLoS Comput Biol. 2023 Jun 22;19(6):e1011227. doi: 10.1371/journal.pcbi.1011227 (PMC10321651; doi:10.1371/journal.pcbi.1011227)

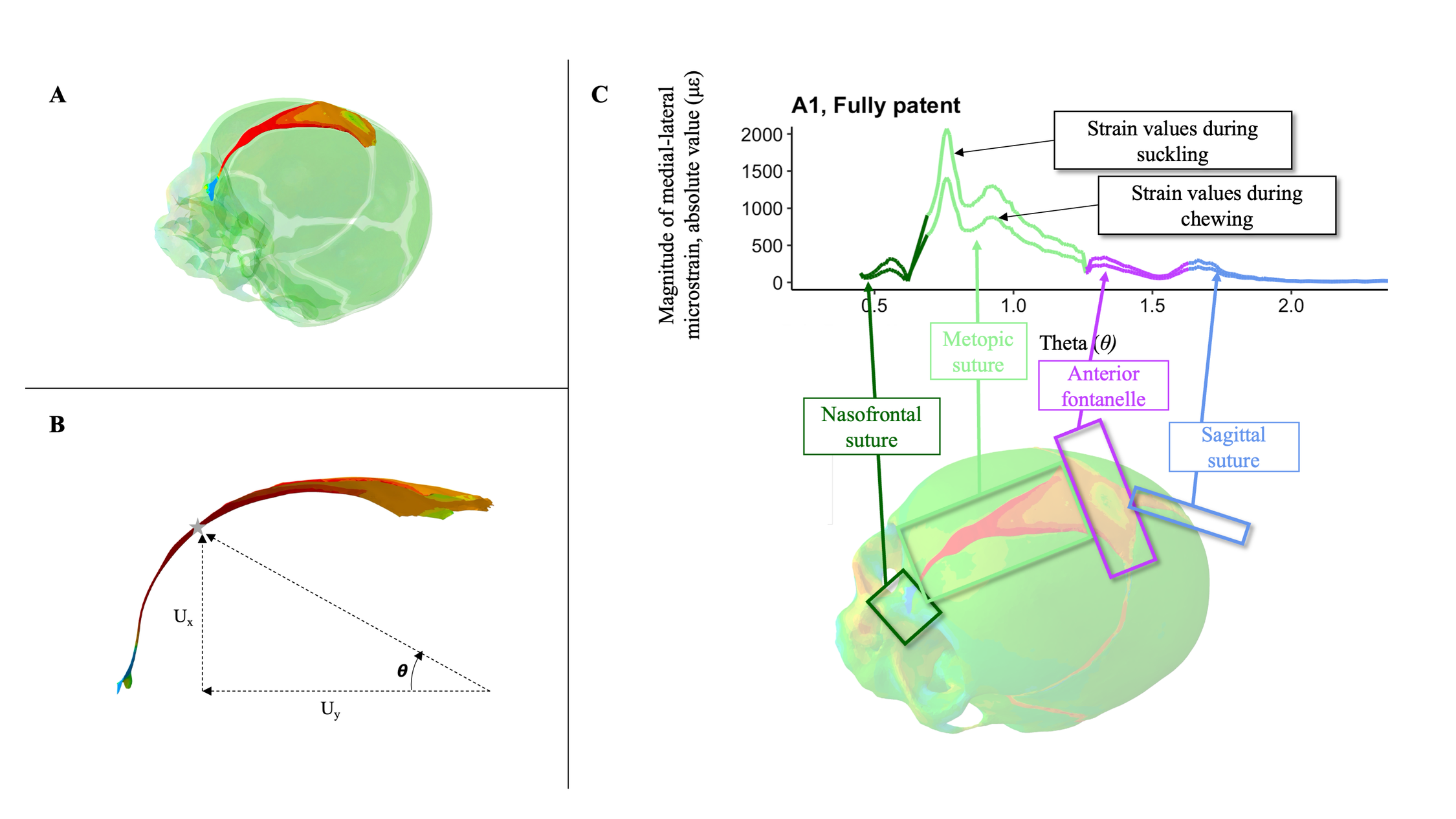

Supplement: S1 Fig — Relevant strain values were isolated for nodes in the sutures along the mid-sagittal plane of the cranial vault, including the nasofrontal, metopic, sagittal, and anterior fontanelle. (Panel A) The coordinate of each node was transformed into a 2-dimensional polar coordinate system with θ defined as arctan(UxUy), with Ux and Uy representing the x and y-coordinates of the node respectively. Note, our coordinate system has the x axis as superior-inferior and the y axis as anterior-posterior. (Panel B) Strain values were grouped and averaged by the θ value, with ranges of θ corresponding to each suture along the mid-sagittal plane. (Panel C). (TIF) [file pcbi.1011227.s003.tif]

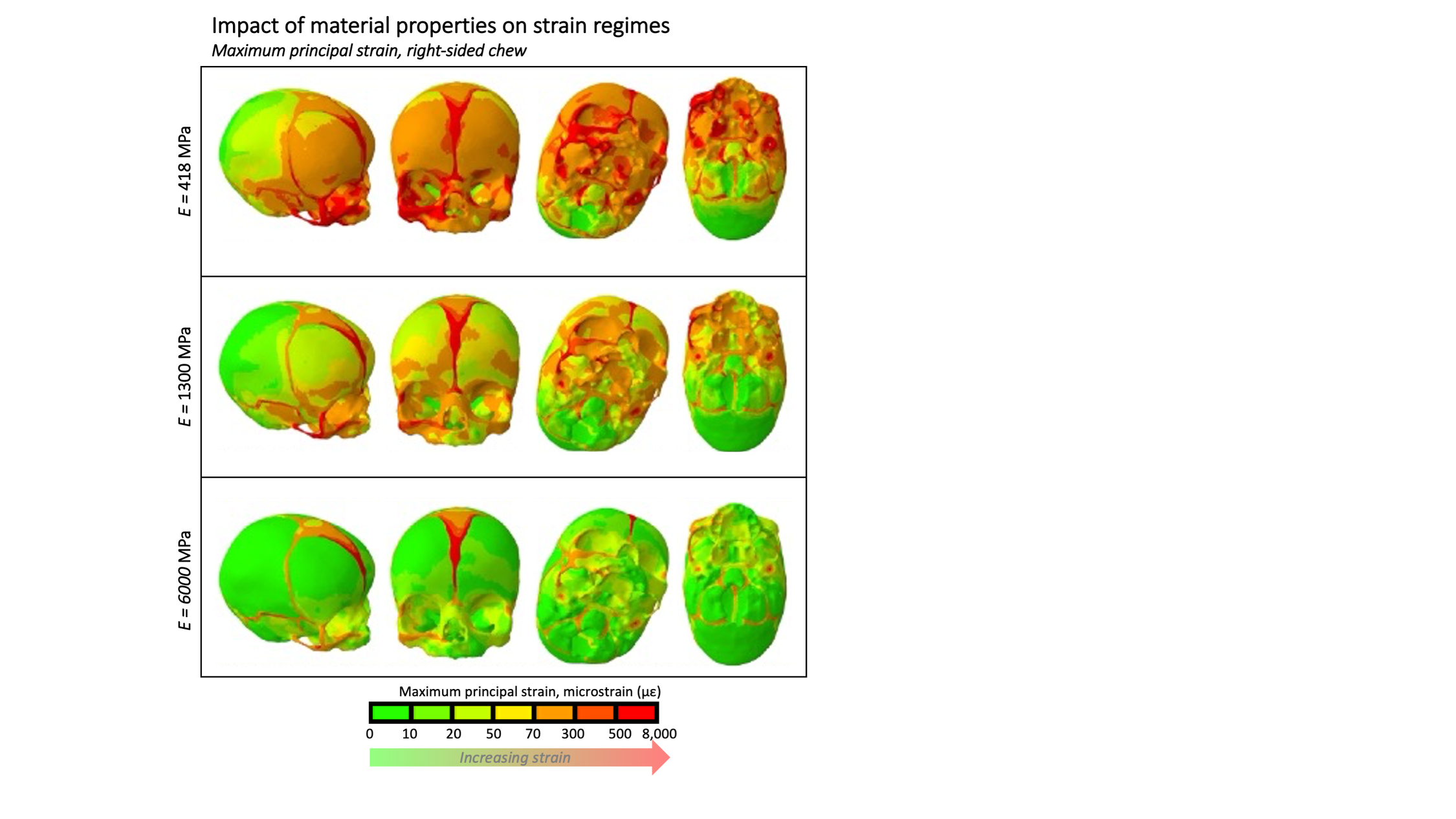

Supplement: S2 Fig — From top to bottom, material properties were: (Top) bone E = 418 MPa; v = 0.27, sutures E = 50 MPa; v = 0.30 | (Middle) bone E = 1300 MPa; v = 0.27, sutures E = 50 MPa; v = 0.30 | (Bottom) bone E = 6000 MPa; v = 0.27, sutures E = 50 MPa; v = 0.30. Increasingly warm colors indicate higher levels of strain. As expected, choosing less stiff material properties (lower E) resulted in higher magnitudes of strain, but the pattern and distribution was relatively unchanged. For example, across the cortical bone, the highest strain values in each model were seen along the right zygomatic arch and right inferior orbital rim, and strain values in the frontal bone were highest near the frontal eminences. (TIF) [file pcbi.1011227.s004.tif]

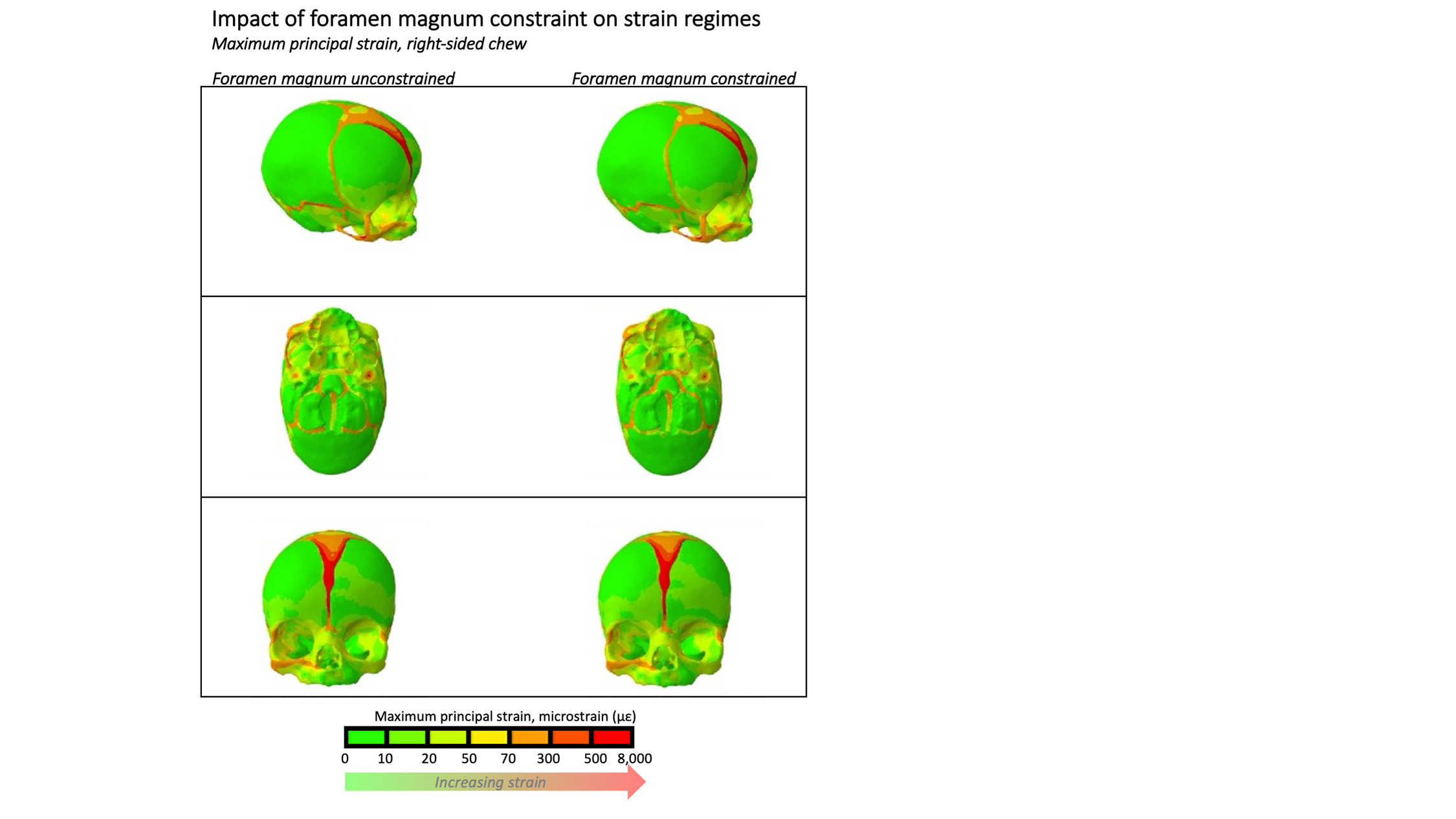

Supplement: S3 Fig — Sensitivity analysis comparing maximum principal strain during right-sided chewing with the foramen magnum constrained in translation along all axes (right) and unconstrained (left). Increasingly warm colors indicate higher levels of strain. Strain magnitudes and patterns across the cranium are nearly identical between the two models, with the only notable difference being increased strain in the inferior skull base sutures bordering the foramen magnum in the constrained model. (TIF) [file pcbi.1011227.s005.tif]

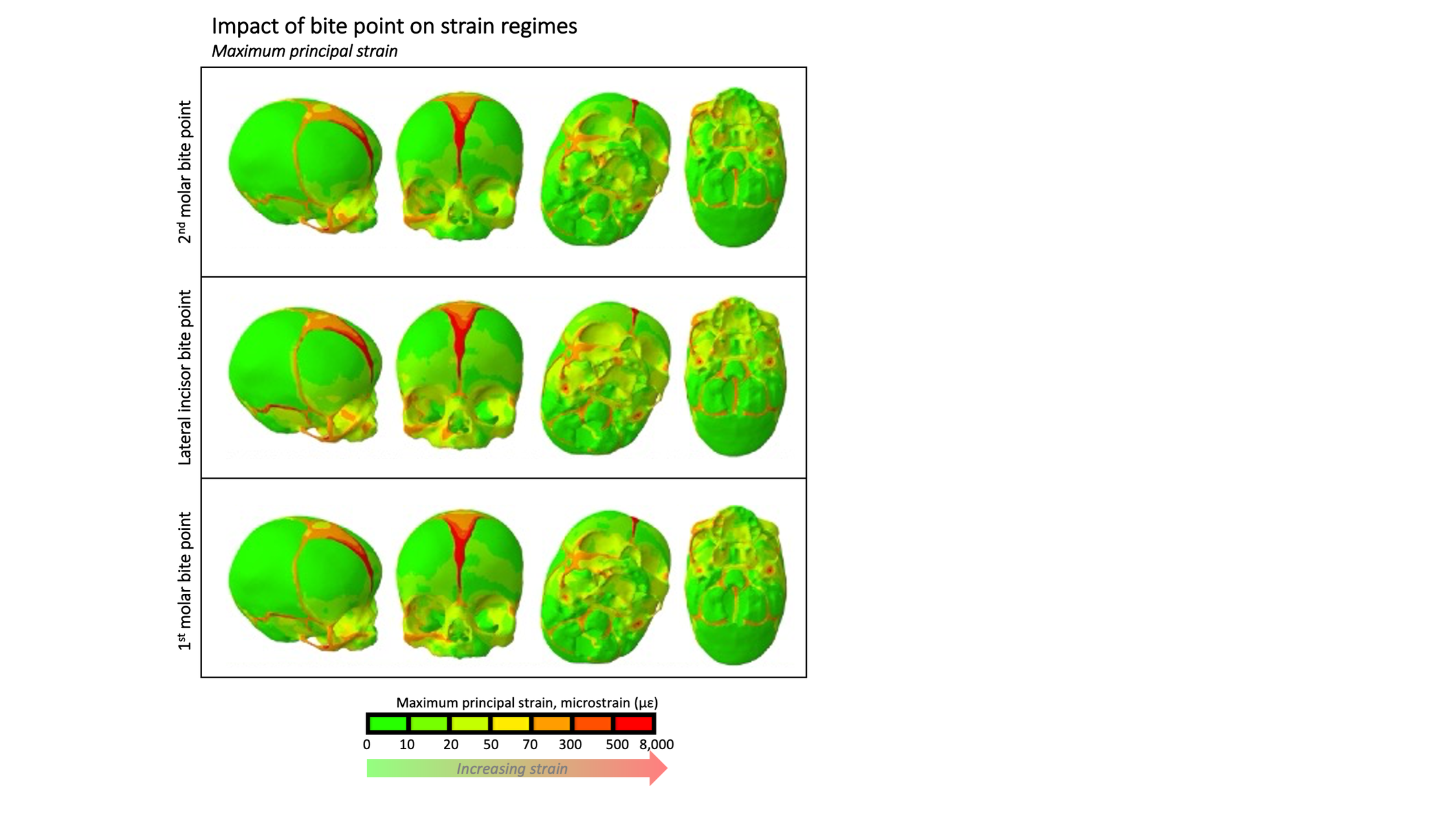

Supplement: S4 Fig — From top to bottom, the bite points are (top) 2nd molar, (middle) lateral incisor, and (bottom) 1st molar. Increasingly warm colors indicate higher levels of strain. The more anterior bite points (lateral incisor and 1st molar) show increased strain along the medial orbital floor and inferior nasal rim, but strain patterns magnitudes across the remaining cranial vault are largely similar. (TIF) [file pcbi.1011227.s006.tif]

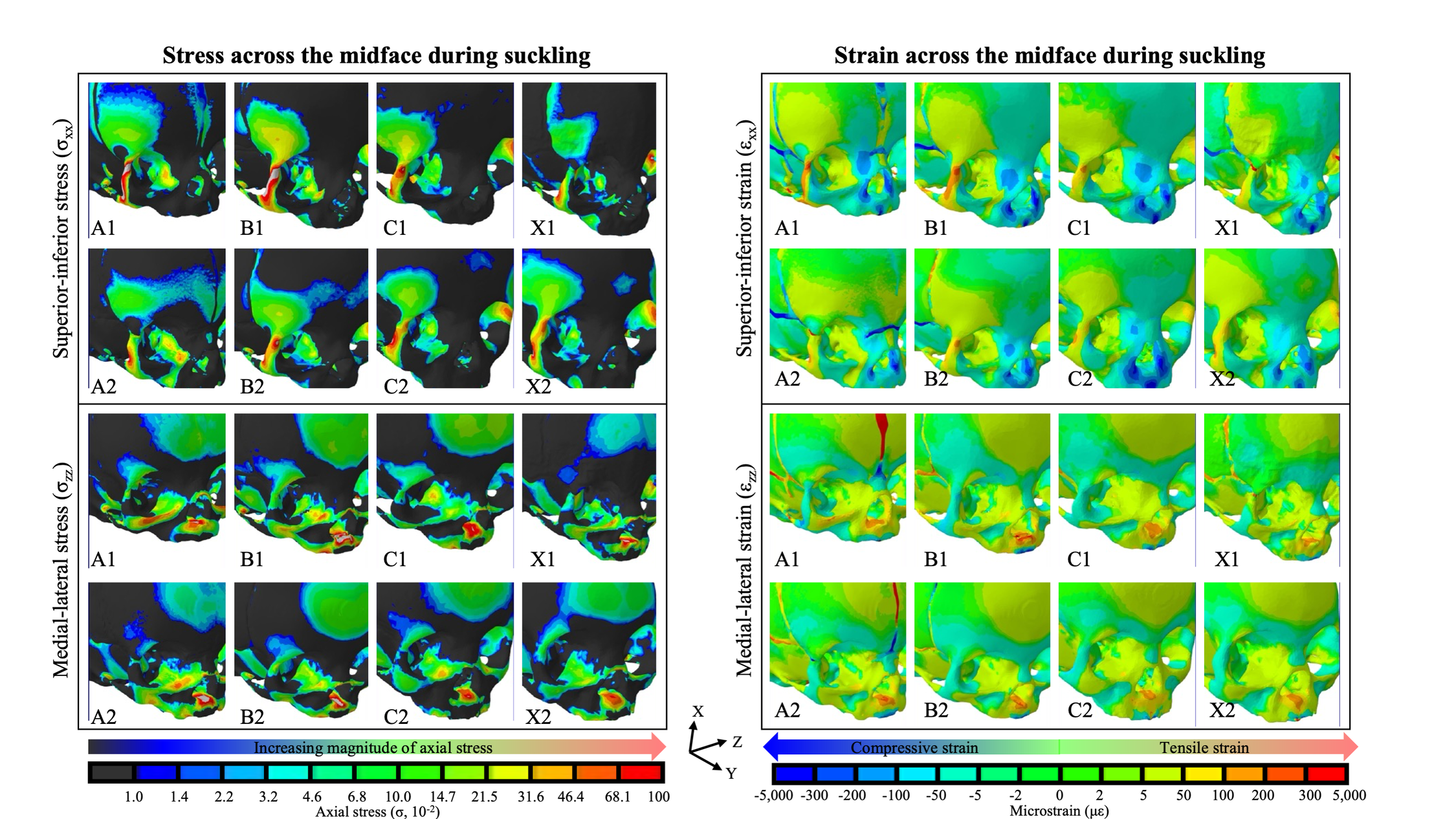

Supplement: S5 Fig — Left panel depicts stress, right panel depicts strain. In all models, superior-inferior stress and strain were highest along the classically understood lateral maxillary vertical buttress, from the zygoma along the lateral orbital wall. The high levels of superior-inferior axial stress along the lateral orbital wall corresponded to tensile (positive, red) superior-inferior strain in the region, with corresponding compressive (negative, blue) superior-inferior strain along the medial orbital wall. Medial-lateral stress and strain were highest along the classically understood transverse maxillary buttress, specifically the lower transverse buttress along the alveolar process and the upper transverse buttress along the inferior orbital rim. Stress and strain following the classically understood maxillary buttresses provides supporting evidence that the models are correctly modeling the physiologic mechanical landscape. (TIF) [file pcbi.1011227.s007.tif]
