# Supplementary material for: The biomechanics of chewing and suckling in the infant: A potential mechanism for physiologic metopic suture closure
Source: PLoS Comput Biol. 2023 Jun 22;19(6):e1011227. doi: 10.1371/journal.pcbi.1011227 (PMC10321651; doi:10.1371/journal.pcbi.1011227)
Supplement: Attachment — Submitted filename: Manuscript Changes_PLOSCompBio.docx [file pcbi.1011227.s008.docx]

Dear Editorial Board of *PLOS Computational Biology*,

In transferring our manuscript from *PLOS Biology* to your journal, we have made minor changes to the manuscript to better fit your journal’s editorial requests as outlined below.

The abstract was shortened to <300 words with the following edits:

- “Animal studies have shown that cyclical strain (e.g. from chewing and suckling) influences the closure of cranial vault sutures, especially the metopic suture, an important locus of craniosynostosis.” -> “Animal studies have shown that cyclical strain from chewing and suckling influences the closure of cranial vault sutures, especially the metopic, an important locus of craniosynostosis.”
- “However, there are no human data on how strain patterns during chewing and suckling might affect, or be affected by, either the physiologically early closure pattern of the metopic suture or pathologic metopic craniosynostosis.” -> “However, there are no human data correlating strain patterns during chewing and suckling with the physiologically early closure pattern of the metopic suture.”
- “Furthermore, differences in craniofacial morphology and development between animals and humans make it challenging to directly extrapolate animal findings to humans.” -> “Furthermore, differences in craniofacial morphology make it challenging to directly extrapolate animal findings to humans.”
- “Muscle forces acting on the cranium during chewing and suckling were simulated using subject-specific jaw muscle physiological cross-sectional areas, and resulting strains and stresses were extracted from the modeled craniofacial skeleton and cranial vault.” -> “Muscle forces acting on the cranium during chewing and suckling were simulated using subject-specific jaw muscle cross-sectional areas.”
- “In models of physiologic suture closure, increased degree of metopic suture closure correlated with higher maximum principal strains across the frontal bone and mid-face, a strain regime not observed in the models of severe metopic craniosynostosis. Our work provides human evidence that bone strain patterns from chewing and suckling correlate with the physiologically early closure pattern of the metopic suture. In isolated metopic craniosynostosis, deviations from physiologic strain regimes at the frontal bone and mid-face may contribute to the clinically observed craniofacial dysmorphism.” -> “In models of physiologic suture closure, increased degree of metopic suture closure correlated with higher maximum principal strains across the frontal bone and mid-face, a strain regime not observed in models of severe metopic craniosynostosis. In summary, our work provides human evidence that bone strain patterns from chewing and suckling correlate with the physiologically early closure pattern of the metopic suture, and that deviations from physiologic strain regimes may contribute to clinically observed craniofacial dysmorphism.”

We also added the “Author Summary” section following the abstract:

Developing infant skulls have multiple “sutures”, which are gaps between the different bones of skull. The sutures fuse together and close at different times, and the proper sequence and timing of closure is important to allow the skull and brain to grow. If the sutures fuse together too early, children can develop a condition called craniosynostosis, which requires intensive surgical intervention to fix. However, understanding the pathologic state necessitates understanding what drives the timing of suture closure in the healthy state. There is limited human data investigating the role of mechanical forces in that process. Our work shows that chewing and suckling in infants causes unique strain patterns conducive to closure across the metopic suture (the suture that closes first in normal development), the first suture to close in normal development. Specifically, the metopic suture experienced larger amounts of compressive and tensile strain with less shearing strain i.e., sideways movement. Along the metopic suture, this strain pattern was most prominent in regions that close first, and least prominent in regions that close last. In short, our work suggests that the forces generated from chewing and suckling may be partially responsible for the timing of metopic suture closure.

Sincerely,

Russell R. Reid, MD PhD

Bernard G. Sarnat Professor of Surgery and Craniofacial Research

Director of Cleft and Craniofacial Services

Residency Program Director

Section of Plastic Surgery

The University of Chicago Medicine & Biological Sciences

5841 S. Maryland Ave. Rm. J-641, MC 6035

Chicago IL 60637

Office: 773-702-6302

Fax: 773-702-1634
