# Supplementary material for: The biomechanics of chewing and suckling in the infant: A potential mechanism for physiologic metopic suture closure
Source: PLoS Comput Biol. 2023 Jun 22;19(6):e1011227. doi: 10.1371/journal.pcbi.1011227 (PMC10321651; doi:10.1371/journal.pcbi.1011227)
Supplement: Attachment — Submitted filename: ResponseToEditors_PLOSCompBioRRR edit.docx [file pcbi.1011227.s009.docx]

Dear Dr. Alber,

We appreciate the reviewers’ questions and edits regarding our article *The biomechanics of chewing and suckling in the infant: a potential mechanism for physiologic metopic suture closure*. We have included our responses to each of the reviewers’ comments and detailed the resulting changes in the manuscript below.

**Reviewer 1**

Authors have used finite element method to quantify the level of mechanical strain across the calvarial sutures in eight individuals modelling chewing and suckling. The study has a particular focus on the metopic suture to comment on a potential mechanism for physiologic fusion of this suture (this would be challenging, not considering the brain growth). Nonetheless, to the best of my knowledge, this is indeed the first study in this field to comment on the level of strains that calvarial sutures are experiencing during infancy as a result of chewing and sucking.

Overall, I find the study novel and informative. It would be great if authors can somehow address the following points in the revised manuscript.

1) Can you please include a figure of all the eight models (A1, A2, B1, B2 etc.) so the reader can visualise the overall morphology of the sutures across the models.

We have added a figure that presents each of the models in multiple views, allowing full visualization of the cranial bone and suture morphology, referenced at L167: “Visualizations of each model to scale are provided in Figure 1...”

**Figure 1.** Visualization of each of the models, to scale, with gray color indicating cranial bone and orange indicating cranial suture. The views presented are, from left to right, front view, vertex view, back view, and right lateral view.

2) Please outline the rough number of elements that were used to mesh the models.

We have added the exact elements per model into supplementary table 1, as well as an approximate number into the main text, L164-168: “…to create non-manifold files with approximately 0.6 to 2.6 million linear tetrahedral elements of 1-2 mm… full details of models are presented in Supplementary Table 1.”

3) In the limitation paragraph of the discussion please address that the radial expansion of the brain during the first year of life was not modelled in this study. And it could be that it is the dynamic between the lowering in the pace of brain growth and the rise in the level of chewing that causes fusion of the metopic suture etc.

The reviewer brings up an insightful and valid consideration here, and we have incorporated this limitation into our discussion section at L658-662: “Finally, we have not modelled intracranial pressure nor radial expansion of the developing brain, which prior studies have linked to cranial vault development. (53) As such we are unable to comment on whether a slowing pace of brain growth, in addition to or in conjunction with, masticatory forces, further contributes to cranial vault suture closure.”

4) Can you please comment/look into the level of principal strains in one of the models e.g. A1 to comment whether metopic suture is predominantly under tension or compression i.e. compare the 1st vs 3rd principal strain in this suture.

We have added a brief summary of the maximum/minimum principal strains along the metopic in models A1 and A2 which highlights that the metopic is predominantly under tension, L372-377: “When considering principal strains, the metopic suture was predominantly under tension, evidenced by larger magnitudes of maximum principal strain (ε1) than minimum principal strains (ε3). Average ε1 along the metopic suture during chewing and suckling in model A1 was 527 με and 763 με respectively, while ε3 was -200 με and -291 με respectively. In model A2, average ε1 along the metopic suture during chewing and suckling was 332 με and 333 με, while ε3 was -162 με and -167 με respectively.”

Specific comments:
L84-94 & L111-112 in relation to the role/impact of mechanical forces on the cranial sutures you may consider following recent studies:
Soh SH, Rafferty K, Herring S. Cyclic loading effects on craniofacial strain and sutural growth in pigs. Am J Orthod Dentofacial Orthop. 2018;154(2):270-282.
Moazen, M., Hejazi, M., Savery, D., Jones, D., Marghoub, A., Alazmani, A., & Pauws, E. Mechanical loading of cranial joints minimizes the craniofacial phenotype in Crouzon syndrome. Scientific reports,2022; 12(1), 9693.

These are both interesting and relevant studies, and we have incorporated them into the introductory section on the role of mechanical forces on suture closure, L90-92: “Interestingly, some in-vivo mice and sheep studies have also shown that cyclic forces can augment cranial vault suture closure by maintaining patency and increasing sutural growth (19,20).”

L141 “…and Table 1. A summarized schematic of the …”

We have incorporated this grammatical change, the sentence in question, now positioned at L286 reads: “…are depicted in Figure 3 and Table 2. A summarized schematic of…”

L495 “…cyclical forces drive suture closure,…” please edit this. I think there is strong body of literature to suggest that, it is the net polarity of the strain in a suture that perhaps dictate its fate i.e. patency or closure.

We agree that this wording was not ideal, and have changed it to read as follows at L668-672: “Given the existing evidence that forces and their resulting strains augment suture closure, our work supports the theory that forces from chewing and suckling contribute to the physiologic closure of the metopic suture in humans, and in the case of trigonocephalic crania, may contribute to some of the clinically observed dysmorphic craniofacial features.”

L519 please comment if the skull base joints were modelled in this study or not? i.e. were synchondroses modelled with soft tissues properties?
We did model the skull base joints, and have now clarified that in the methods section at L157-163: “Patient CT scans were segmented in Mimics v21.0 (Materialise NV, Belgium) to separate cranial bone, sutures, skull base synchondroses, and the mandible… The synchondroses of the skull base were modeled using the same material properties as the cranial vault sutures.”

L534 “Muscle forces were estimated using….”

This edit has been incorporated into L182, which now reads as: “Muscle forces were estimated using model-specific estimates of muscle…:

L561 “…measured from human unilateral chewing for the superficial…” this statement in the light of L142-143 is abit confusing i.e. were the results presented in L137 caused by bilateral contraction of the specified muscle groups or unilateral contraction.

We agree that this wording is confusing, and have changed the section in question to now read as follows at L207-212: “Force magnitudes for each muscle were calculated by multiplying the model-specific muscle CSA with a specific tension of 22.5 N/cm^2^ and electromyographic (EMG) activation factors measured from human chewing for the superficial and deep masseter, anterior and posterior temporalis, and the medial pterygoid (44,48). The chewing condition modeled includes bilateral muscle activation, with the right-side functioning as the working side and the left-side functioning as the balancing side.”

L588 please introduce a separate section and call it “Material properties” there detail the material properties that were used on the base model which its results are described in the main text. I appreciate that these are outlined in the Sensitivity Analysis section (L606) but i think it is more appropriate to separate the two to avoid any confusion as per choice of the material properties that were used to generate the results described e.g. in L138-158.

This is a valid call-out, we have added a Material properties section into the methods, which now reads as follows at L238-241: “Isotropic and homogeneous material properties were assigned to the bones of the cranial vault (E=6000 MPa; v=0.27) and sutures (E=50 MPa; v=0.30) based on the prior work of Jasinoski et al (51). These material properties were used for all models and data presented in our results section.”

We also changed the wording of the sensitivity analysis section to read as follows at L264-268: “The impact of material properties on model behavior was assessed first. Two additional sets of material properties based on the work of Borghi et al. (bone, *E*=418 MPa) and Barbeito-Andres et al. (bone, *E*=1300 MPa) were simulated and resulting maximum principal strain regimes were compared to the results that utilized material properties from Jasinoski et al. (bone, *E*=6000 MPa), as detailed in Supplementary Figure 2 (52,53).”

L598 “…average nodal strain from the endo- and ectocranial surface nodes of the suture.” Can you please clarify this further i.e. were the average values reported per sutures calculated across the whole volume of the sutural elements or only their surface values (both on the endo and ectocranial).

The average values reported were only from the surface endo/ectocranial values, not the whole volume; this clarification has now been added in, and L253-255 now reads as: “These strain values, as well as the other axial and shear strains, were calculated as the average nodal strain from the surface nodes of the endo- and ectocranial surfaces of the suture.”

**Reviewer #2:**

I had the privilege to review this article submitted for publication to PLOS Computational Biology.
In this paper, finite element modelling is used to estimate suture strains during suckling and chewing in paediatric subjects aged 3 months - 3 years with different levels of metopic suture fusion.
This is an extremely well designed study and the results show promising correlation between suture closure and strain levels. The methodology is very well described and the results are presented reasonably clearly, considering the amount of information is conveyed.
I have very little to comment on an otherwise already very good manuscript.

- The authors may consider to reshuffle the paragraphs and have the methodology as second section to make the manuscript more readable (I had to read it this way to understand)

We tend to agree and have switched the methods to the second section. We acknowledge that the PLOS Computational Biology standard format has the methods section at the end but believe that in this article understanding the methodology prior to diving into the results and discussion helps to better convey the information. The new methodology section begins at L137

- The author should comment on the patient selection and state the reasons why the normal subjects received CTs.

We have added this information into the patient selection section of the methodology as follows at L146-151: “Of the physiologically normal patients, 4 received CT scans due to concern for early closure of the anterior fontanelle or abnormal head shape but were found have patent sutures and fontanelles, 1 received a CT scan due to a concern for hemorrhage following trauma but was found to have no evidence of intracerebral bleeding or osseous damage, and 1 received a CT scan due to concern for a stroke secondary to a sickle cell attack but was found to have no intracerebral anomalies.”

- The authors should show frontal, lateral and top view of all the 8 patients, possibly in scale to appreciate also differences in size

We have added an additional figure, Figure 1, which includes to scale views of each of the patients.

- How were the axis oriented? I presume the frankfurt plane was assumed horizontal. The selection of the origin affects the results in figure 7, you may want to state how it was chosen.

Details regarding the orientation of the axes, location of the origin, and orientation of the planes have been added into the methodology section at L171-174: “Axes were oriented as follows: the (+/-) x-axis was defined as superior/inferior, the (+/-) y-axis was defined as anterior/posterior, and the (+/-) z-axis was defined as left/right mediolateral. The origin was chosen as the midpoint between the condyles of the mandible, and the horizontal x-z plane was defined as the Frankfurt plane.”

- The authors simplify the strain analysis of the strain actoss a suture using the axial strain perpendicular to the main direction of the suture. Although this is a reasonable simplification, the authors should consider to provide some indication of the error introduced by this assumption.

This is a valid critique of the simplification, and we have amended the section detailing the model analysis methods to read as follows at L246-249: “. Strain across a suture (i.e. that would contribute to oscillatory compression and tension) was defined as axial strain in the axis perpendicular to the main direction of the suture. This simplification facilitates comparison of strain between sutures and is broadly accurate, but it does not capture the impact of local deviations in suture orientation.”

- The authors should comment on some of the intra-subject variability: although a clear trend is visible, patients A1 and A2 have relatively different strain levels shown in figure 5 and figure 6. Does the age of patient C2 have an effect on the results?

These observations are accurate, and the differences between A1 (fully patent) and A2 (fully patent, but narrowing) are in-line with the overall trend that strain magnitudes across and along the metopic tend to decrease as it progresses towards closure. This has been added to the manuscript in two spots:

First, at L383-386: “Between the two models with a fully patent metopic suture, the model with the narrower metopic suture (A2) tended to have a lower magnitude of axial and shear strain across the suture, in line with the trend of lower strain magnitudes across and along the suture as it progresses towards closure.”

And then again at L431-434: “Relative to the fully patent A1 model, ε_zz_ strain magnitudes along the A2 model tended to be lower in magnitude, consistent with the observed trend of decreased strain magnitudes along and across the metopic suture as it progressed towards closure.”

From our interpretation of the results, the age of C2 does not appear to have had an effect on the results, as the strain and stress values throughout tend to agree closely with C1. We have commented on this with an addition into the discussion section at L571-576: “Third, one might have expected a larger difference in strain and stress distributions in the C2 model given the relatively older age of 3 years and larger size of the cranium. However, compared to C1, the other fully physiologically closed metopic suture model of 7 months, minimal differences in strain and stress values across the cranial vault sutures or cranium were noted. This could be a result of the larger muscle mass in C2 being neutralized by the increased mass of the cranial vault sutures, or merely too small of a sample size to ascertain a difference.”

- How does the current analysis relate to the pathological closure of the metopic suture in craniosynostosis: suture closure has been reported to start in the second trimester and trigonocephaly has been reported as early as at 20 weeks post-conception
([https://doi.org/10.1080/14767058.2017.1335706](https://urldefense.com/v3/__https:/doi.org/10.1080/14767058.2017.1335706__;!!MvNZe7V6M35iZPhbgng-hfU!zyuRzdfCjfmG1fyLmx3rNvn7_5KHkJybs1HQt-MbrVUKFe6Z_lMBtaADNjIPqjUvDP4w9oardx8ez8BqERhZ$)), can you comment on this in light of your results?

This is an insightful question, and we have commented on this in the discussion section as follows at L602-607: “Interestingly, pre-mature pathologic metopic suture closure has been reported to begin in the second trimester, well before one might expect chewing and suckling to be the primary biomechanical forces at play. (69,70) However, our results support the hypothesis that physical forces play a role in physiologic closure, and that lends support to the hypotheses that physical forces experienced in-utero may play a part in early pathologic metopic suture closure, as evidence has shown that metopic craniosynostosis is not solely a result of genetics. (71)”

Thank you again for considering our manuscript for publication, and we look forward to hearing from you with next steps.

Sincerely,

Russell R. Reid, MD PhD

Bernard G. Sarnat Professor of Surgery and Craniofacial Research

Director of Cleft and Craniofacial Services

Residency Program Director

Section of Plastic Surgery

The University of Chicago Medicine & Biological Sciences

5841 S. Maryland Ave. Rm. J-641, MC 6035

Chicago IL 60637

Office: 773-702-6302

Fax: 773-702-1634
